# Gut microbiota regulation of T lymphocyte subsets during systemic lupus erythematosus

**DOI:** 10.1186/s12865-024-00632-0

**Published:** 2024-07-08

**Authors:** Fen-Ping Lian, Fen Zhang, Chun-Miao Zhao, Xu-Xia Wang, Yu-Jie Bu, Xing Cen, Gui-Fang Zhao, Sheng-Xiao Zhang, Jun-Wei Chen

**Affiliations:** 1 Department of Rheumatology, the Second Hospital of Shanxi Medical University, Taiyuan City, Shanxi Province 030001 China; 2 Department of Rheumatology, Xi’an International Medical Center Hospital, Xi’an City, Shaanxi Province China

**Keywords:** Systemic lupus erythematosus, Gut microbiota, T lymphocyte subsets

## Abstract

**Background:**

Systemic lupus erythematosus (SLE) is an autoimmune disease characterized by disturbance of pro-inflammatory and anti-inflammatory lymphocytes. Growing evidence shown that gut microbiota participated in the occurrence and development of SLE by affecting the differentiation and function of intestinal immune cells. The purpose of this study was to investigate the changes of gut microbiota in SLE and judge its associations with peripheral T lymphocytes.

**Methods:**

A total of 19 SLE patients and 16 HCs were enrolled in this study. Flow cytometry was used to detect the number of peripheral T lymphocyte subsets, and 16 s rRNA was used to detect the relative abundance of gut microbiota. Analyzed the correlation between gut microbiota with SLEDAI, ESR, ds-DNA and complement. SPSS26.0 software was used to analyze the experimental data. Mann–Whitney U test was applied to compare T lymphocyte subsets. Spearman analysis was used for calculating correlation.

**Results:**

Compared with HCs, the proportions of Tregs (*P* = 0.001), Tfh cells (*P* = 0.018) and Naïve CD4 + T cells (*P *= 0.004) significantly decreased in SLE patients, and proportions of Th17 cells (*P* = 0.020) and γδT cells (*P* = 0.018) increased in SLE. The diversity of SLE patients were significantly decreased. Addition, there were 11 species of flora were discovered to be distinctly different in SLE group (*P* < 0.05). In the correlation analysis of SLE, Tregs were positively correlated with *Ruminococcus2* (*P* = 0.042), Th17 cells were positively correlated with *Megamonas* (*P* = 0.009), γδT cells were positively correlated with *Megamonas* (*P* = 0.003) and *Streptococcus* (*P* = 0.004), Tfh cells were positively correlated with *Bacteroides* (*P* = 0.040), and Th1 cells were negatively correlated with *Bifidobacterium *(*P* = 0.005). As for clinical indicators, the level of Tregs was negatively correlated with ESR (*P* = 0.031), but not with C3 and C4, and the remaining cells were not significantly correlated with ESR, C3 and C4.

**Conclusion:**

Gut microbiota and T lymphocyte subsets of SLE changed and related to each other, which may break the immune balance and affect the occurrence and development of SLE. Therefore, it is necessary to pay attention to the changes of gut microbiota and provide new ideas for the treatment of SLE.

**Supplementary Information:**

The online version contains supplementary material available at 10.1186/s12865-024-00632-0.

## Introduction

Systemic lupus erythematosus (SLE) is a common chronic autoimmune disease involving multiple organs and systems, which leads to diverse clinical manifestations. The pathogenesis of SLE is not entirely clear, and may be related to various factors such as genetic, immune, hormones, and environment [1]. Pathogenic factors stimulate the generation of autoantibodies and activation of autoreactive T cells, resulting in abnormal immune environment [2]. To a certain extent, immunosuppressive drugs could correct immune disorder and reduce disease activity. However, there are still some SLE patients who are not in remission, and long-term medication may increase the risk of severe infection and death [3]. Therefore, we need to better understand the immune-related pathogenesis of SLE in order to optimize the treatment strategies.

In recent years, numerous studies have highlighted the imbalance of T lymphocyte subsets in the onset of both lupus murine and SLE patients [4, 5]. In addition, the regulation of T subpopulations by gut microbiota and their metabolites in SLE sufferers are also widely recognized [6]. Oral antibiotics could suppress the growth of bacteria and induce autoimmune diseases such as SLE, which suggested the critical role of bacteria balance in SLE [7]. It has been shown that changes in gut microbiota, particularly those of *Clostridium* and *segmented filamentous bacteria (SFB)* in mice and *Bacteroides fragilis* in humans can disrupt the homeostasis between Type 17 T helper (Th17) and T regulatory (Treg) cells (Tregs) in autoimmune diseases [8]. Moreover, a study has increased the proportion of *Lactobacillales* in gut microbiota, thus stimulating differentiation of Tregs and inhibiting differentiation of pathogenic Th17 cells to alleviate renal inflammation in lupus-prone mice, which suggests another potential treatment for SLE [9].

The development of metagenomic sequencing (16S rRNA and whole-genome sequencing) has made it possible to study the mechanisms of the gut microbiota in many diseases. Here, we report changes of gut microbiota and T lymphocyte subpopulations in SLE patients as well as the correlation between them to explore the pathogenesis for more effective treatment.

## Materials and methods

### Participants

A total of 19 patients with SLE (mean age of 42.79 ± 14.36 years old) according to the 2019 American college of Rheumatology (ACR) and European League Against Rheumatism (EULAR) classification criteria were included in the study. At the same time, 16 healthy controls (HCs) (mean age of 33.62 ± 12.63 years old) were also enrolled in this research. All participants donated samples of their peripheral blood (PB) and feces for the test of T lymphocyte subsets and intestinal microflora respectively. Clinical data like erythrocyte sedimentation rate (ESR), complement 3 (C3) and complement 4 (C4) were recorded in detail. None of the selected members had serious heart, liver, kidney diseases, tumor, infection, use of antibiotics within two months or gastrointestinal surgery. In addition, none of the selected members used probiotic supplements. Each participant signed the informed consent and our study was approved by Ethics Committee of Second Hospital of Shanxi Medical University (Ethics Number: 2023YX246).

### Flow cytometry

Flow cytometry was used to value the plasma levels of T lymphocyte subsets. Surface antibodies, intracellular antibodies were added to label the T cells, including CD4^+^T (CD3^+^CD4^+^), CD8^+^T (CD3^+^CD8^+^), Naïve CD4^+^T (CD3^+^CD4^+^CD45RA^+^), Memory CD4^+^T (CD3^+^CD4^+^CD45RO^+^), Naïve CD8^+^T (CD3^+^CD8^+^CD45RA^+^), Memory CD8^+^T (CD3^+^CD8^+^CD45RO^+^), Tfh (CD4^+^CXCR5^+^PD1^+^), Tfr (CD4^+^CXCR5^+^CD25^+^Foxp3^+^), Th1 (CD4^+^IFN-γ^+^), Th17 (CD4^+^IL-17^+^), Tregs (CD4^+^CD25^+^FoxP3^+^) and γδ T (CD3^+^TCR γδ ^+^).

### DNA extraction from fecal specimens

500 mg fresh stool samples without urine or blood stains from all participants were collected. The samples should be put into pre-prepared storage tubes quickly and transported to laboratory to be frozen and stored at -80 °C for later use. Total DNA were extracted from thawed specimens using the TIANamp Stool DNA Kit according to the manufacturer protocols. The V3-V4 regions of the bacterial 16S rRNA gene sequences were amplified from the diluted DNA extracts with the primers (F-ACTCCTACGGGAGGCAGCAG) and (R-GGACTACHVGGGTWTCTAAT).

### PCR amplification and sequencing

PCR amplification was performed in a mixture containing 2 μl of 10 × Buffer, 5 μl of target DNA sample, 1.5 μl of forward primer, 1.5 μl of reverse primer, 0.5 μl of dNTPs, 0.5 μl of magnesium ion, 0.5 μl of Taq DNA Polymerase and added dH_2_O to 20 μl. The reactions were hot-started at 95˚C for 3 min, followed by 27 cycles at 95˚C for 30 s, 55˚C for 30 s and 72˚C for 60 s, with a final extension step at 72˚C for 7 min. Target PCR products were detected by electrophoresis in 2% agarose gel. The products were purified and recovered by FC magic beans Kit (enlighten) and quantify by Qubit 4.0 (Thermo Fisher Scientific). The Illumina PE250 library was constructed by mixing the samples of equal volume and denatured them with NaOH. Each sample was sequenced on Miseq PE250 (Illumina).

### Bioinformatics analysis: species diversity and difference analysis

Sequence reads were filtered to remove low-quality reads, including those that lack exact match with primers, contain ambiguous characters or average quality score of less than 25. High-quality reads are spliced into tags through overlap between reads. The tags with 97% similarity or more were grouped into the same operational taxonomic units (OTUs). Alpha diversity indices (richness, Chao1, Simpson, Shannon) and Beta diversity indices were analyzed using QIIME2 and results are displayed using R software.

### Statistical analysis

The collected data were examined using SPSS 26.0. For measurement data such as proportions of T subsets and age, Kolmogorov–Smirnov test and Levene's T test were used to test the normality and homogeneity of variance of all variables respectively. Data conforming to normal distribution were described as mean ± standard deviation ($\overline{x} \pm s$) and analyzed by independent-samples *t-*test. Meanwhile, values that didn’t meet the normal distribution were represented as quartile and analyzed by Mann–Whitney U test. For enumeration data like gender, the Chi-square test was applied for statistical analysis. Moreover, Spearman test was used for testing the correlation between two variables. All *P*-values reported herein are two-tailed and *P*-value < 0.05 was taken as statistically significant.

## Results

### Demographic and clinical characteristics of participants

The mean age of 19 SLE patients was 42.79 ± 14.36 years old (female/male:15/4) and that of 16 HCs was 33.62 ± 12.63 years old (female/male:6/10). There were no statistically differences in age and gender between SLE patients and HCs (*P* > 0.05) (Table 1). Furthermore, clinical characteristics like erythrocyte sedimentation rate (ESR), complement 3 (C3), complement 4(C4) have been shown in Table 1.

**Table 1:** Demographic and clinical characteristics of participants ($\overline{x} \pm s$)

	Number	Gender		Age (year)	ESR (mm/h)	C3 (g/L)	C4 (g/L)
		Male	Female				
SLE	19	4	15	42.79 ± 14.36	54.74 ± 40.53	0.53 ± 0.27	0.12 ± 0.10
HC	16	10	6	33.62 ± 12.63	-	-	-
*P*		0.118		0.226	-	-	-

### The distribution of T lymphocyte subsets in peripheral blood is unbalanced in SLE patients

**Fig. 1:**
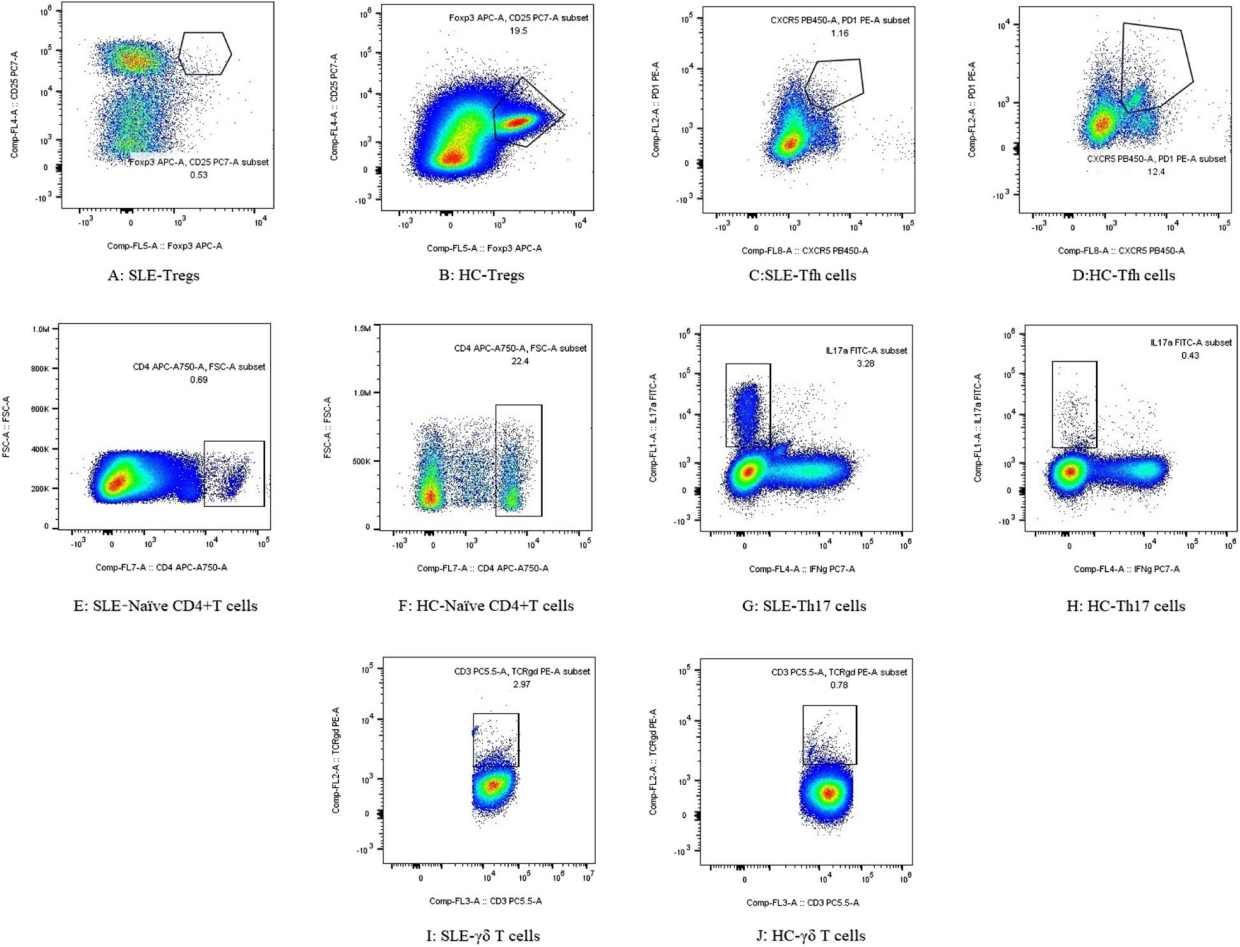
Flow graphs of T subsets with statistically significant differences between SLE patients and HC. **A**, **B** Flow graph of Tregs. **C**, **D** Flow graph of Tfh cells. **E**, **F** Flow graph of Naïve CD4 + T cells. **G**, **H** Flow graph of Th17 cells. **I**, **J** Flow graph of γδ T cells

**Fig. 2:**
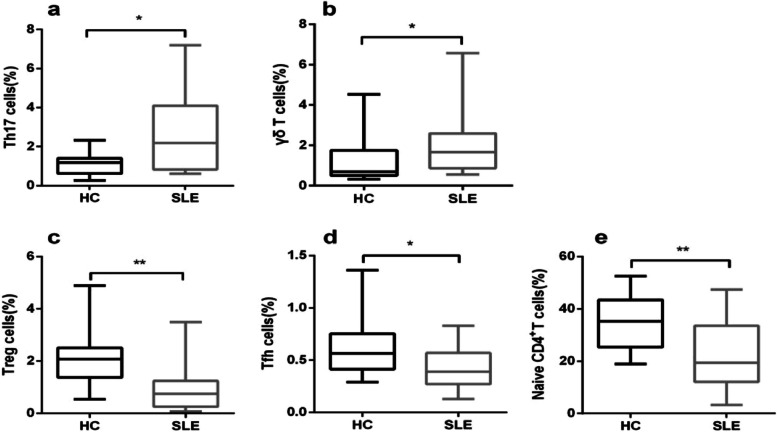
Comparison of 5 T subsets with statistically significant differences between SLE patients and HCs

We tested the distribution of T lymphocyte subsets in peripheral blood using flow cytometry and flow chart is detailed in Fig. 1, the specific gating strategy of T lymphocyte subsets are shown in Figure S1-Figure S10 of the supplementary material. And the statistically differences of results were summarized in Fig. 2.

In particular, the percentages of Th17 cells [2.19 (0.83–4.10) vs. 1.18 (0.62–1.40), respectively, *P* = 0.020] and γδ T cells [1.66 (0.86–2.59) vs. 0.69 (0.50–1.75), respectively, *P* = 0.018] were higher in SLE patients than those in HCs group (Fig. 2a, b). In contrast, compared with HCs, SLE sufferers had lower proportions of Tregs [0.75 (0.25–1.24) vs. 2.08 (1.37–2.51), respectively, *P* = 0.001], Tfh cells [0.39 (0.27–0.57) vs. 0.57 (0.42–0.75), respectively, *P* = 0.018] and Naïve CD4^+^T cells [19.35 (12.12–33.50) vs. 35.24 (25.38–43.41), respectively, *P* = 0.004] (Fig. 2 c-e)*.*

However, there were no significant statistical differences in proportions of CD4^+^T [19.88 (15.39–31.00) vs. 23.05 (18.84–33.48), respectively, *P* = 0.635], memory CD4^+^T [49.27 (34.37–60.78) vs. 35.57 (32.48–47.88), respectively, *P* = 0.109], CD8^+^T [27.35 (20.87–31.88) vs. 23.89 (21.29–27.44), respectively, *P* = 0.367], Naïve CD8^+^T [67.22 (37.89–74.34) vs. 68.94 (60.76–75.04), respectively, *P* = 0.403], memory CD8^+^T [17.37 (13.79–22.52) vs. 13.99 (11.33–25.44), respectively, *P* = 0.350], Tfr [0.13 (0.05–0.16) vs. 0.06 (0.04–0.14), respectively, *P* = 0.109] and Th1 [11.40 (8.07–26.50) vs. 16.25 (11.68–19.05), respectively, *P* = 0.961] between SLE patients and HCs.

### ESR was negatively correlated with the proportion of Tregs

There was a significant negative correlation between Tregs and ESR (*P* = 0.031) in SLE patients, suggesting that ESR decreased with the increase of Tregs. And the values of other peripheral T lymphocyte subsets with statistically differences had no correlation with ESR, C3 and C4 (Table 2).

**Table 2:** The correlation between T subsets with statistically differences and disease activity indicators

	ESR		C3		C4	
	*r*	*P*	*r*	*P*	*r*	*P*
Naïve CD4^+^T	0.128	0.602	-0.060	0.808	-0.083	0.737
Tfh	-0.184	0.451	-0.062	0.802	-0.237	0.328
Th17	-0.146	0.550	-0.109	0.657	-0.009	0.970
Tregs	**-0.495**	0.031^*^	-0.038	0.878	-0.119	0.628
γδ T	-0.044	0.858	-0.330	0.167	-0.273	0.259

^*^P＜0.05

### Variation of gut microbiota in SLE patients compared with HCs

**Fig. 3:**
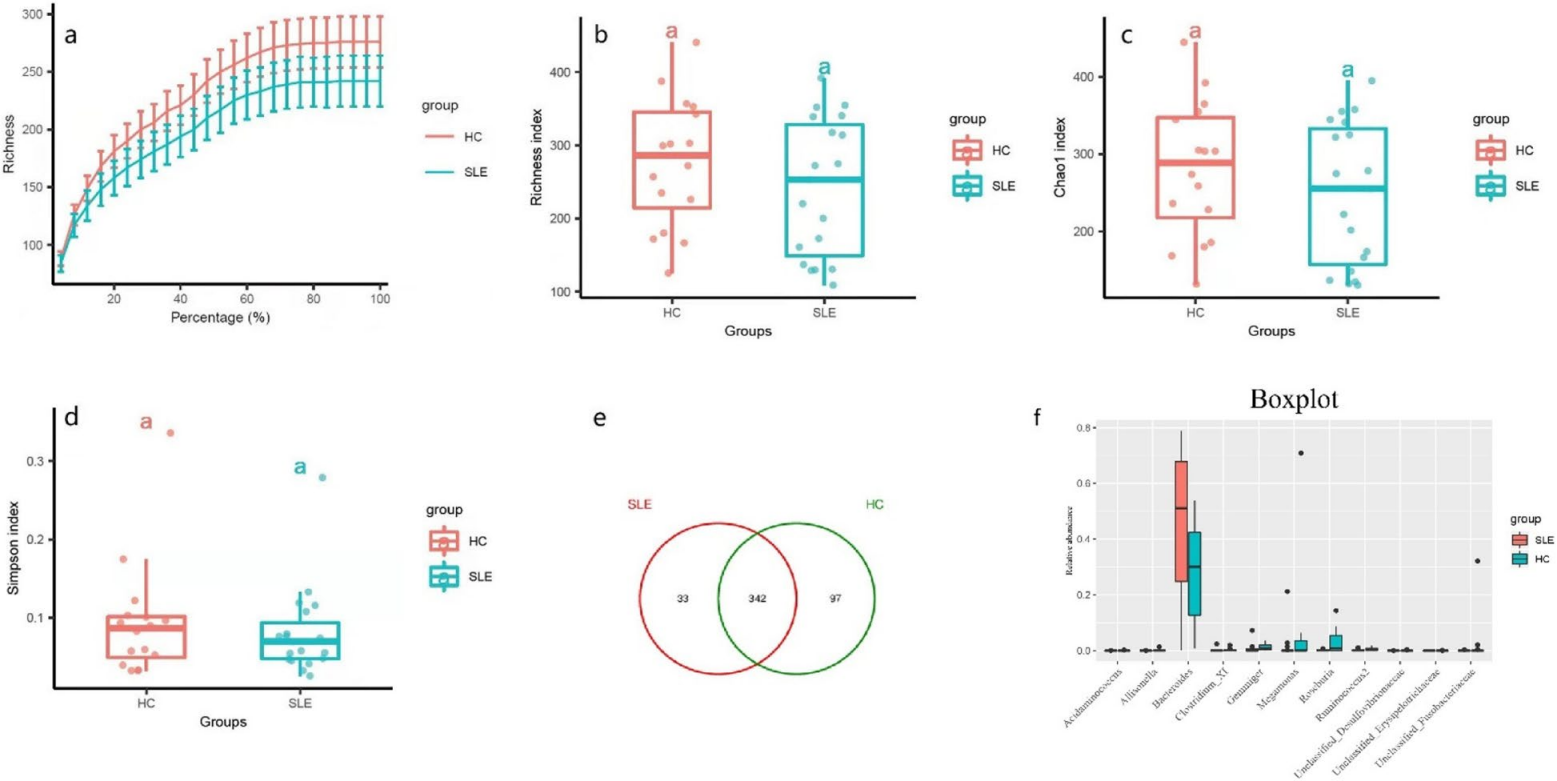
The species richness and evenness of gut microbiota

We examined the alpha diversity of each sample to reflect the species richness and evenness of gut microbiota. The results showed that SLE patients had reduced community richness and obvious quantity differences in every species based on the richness index, Chao1 index and Simpson index (Fig. 3a-d). The Venn diagram that reflecting distribution of OTU clusters indicated 342 OTUs were shared, 97 OTUs were specific to HCs and only 33 were specific to SLE group (Fig. 3e). Compared with HC, 11 species of flora were discovered to be distinctly different (*P* < 0.05) from the boxplot diagram (Fig. 3f). These data clearly indicated a decreased richness of gut microbiota in SLE sufferers and suggested that certain microbiological changes may be associated with the pathogenesis of SLE.

### The relevance between highly expressed differential flora and T peripheral lymphocyte subpopulations in SLE group

As is shown in Table 3, there were significant positive correlations between levels of Tregs and *Ruminococcus2* (*P* = 0.042), Th17 cells and *Megamonas* (*P* = 0.009), γδT cells and *Streptococcus* (*P* = 0.004) as well as *Megamonas* (*P* = 0.003), Tfh cells and *Bacteroides* (*P* = 0.040). However, Th1 cells and *Bifidobacterium* were negatively correlated in these participants (*P* = 0.005).

**Table 3:** The relevance between highly expressed differential flora and T lymphocyte subpopulations in SLE group

	Tregs		Th17 cells		Th1 cells		γδ T cells		Tfh cells	
	r	*P*	r	*P*	r	*P*	r	*P*	r	*P*
Unclassified_Lachnospiraceae	-0.191	0.433	-0.081	0.742	-0.091	0.710	0.285	0.237	-0.115	0.639
Unclassified_Ruminococcaceae	-0.035	0.887	0.050	0.839	-0.453	0.052	0.225	0.355	0.088	0.720
Clostridium_XVIII	0.225	0.355	0.036	0.884	-0.044	0.858	0.374	0.115	-0.221	0.364
Streptococcus	0.000	1.000	0.152	0.535	0.068	0.781	**0.629**	0.004^**^	-0.237	0.328
Bifidobacterium	-0.351	0.141	-0.052	0.833	**-0.615**	0.005^**^	0.304	0.206	-0.137	0.575
Bacteroides	-0.009	0.972	-0.172	0.481	0.011	0.966	-0.407	0.084	**0.475**	0.040^*^
Ruminococcus2	**0.470**	0.042^*^	0.037	0.882	-0.203	0.405	0.160	0.514	0.109	0.658
Megamonas	0.040	0.871	**0.583**	0.009^**^	0.193	0.427	**0.639**	0.003^**^	0.137	0.576

^*^P＜0.05

^**^P＜0.01

### The relevance between highly expressed differential flora and T peripheral lymphocyte subpopulations in HC group

As is shown in Table 4, there were significant negative correlations between Treg levels and *Bacteroides* (*P* = 0.033), Th1 cells and *Streptococcus* (*P* = 0.006), γδT cells and *Ruminococcus2* (*P* = 0.008).

**Table 4:** The relevance between highly expressed differential flora and T lymphocyte subpopulations in HC group

	Tregs		Th17 cells		Th1 cells		γδ T cells		Tfh cells	
	r	*P*	r	*P*	r	*P*	r	*P*	r	*P*
Unclassified_Lachnospiraceae	0.279	0.295	0.125	0.644	-0.044	0.871	-0.150	0.579	-0.003	0.991
Unclassified_Ruminococcaceae	-0.087	0.748	-0.422	0.104	0.021	0.940	0.076	0.778	-0.077	0.778
Clostridium_XVIII	-0.195	0.470	0.121	0.656	0.265	0.322	-0.044	0.871	0.296	0.266
Streptococcus	-0.062	0.820	-0.348	0.187	**-0.650**	0.006^**^	0.103	0.704	-0.115	0.672
Bifidobacterium	0.332	0.209	-0.396	0.128	-0.388	0.137	0.288	0.279	-0.043	0.875
Bacteroides	**-0.534**	0.033^*^	0.133	0.624	0.165	0.542	-0.085	0.753	0.010	0.970
Ruminococcus2	-0.114	0.675	0.464	0.070	-0.071	0.795	**-0.635**	0.008^**^	-0.244	0.361
Megamonas	-0.095	0.726	-0.496	0.051	-0.022	0.935	0.206	0.444	0.242	0.367

^*^P＜0.05

^**^P＜0.01

### C4 was negatively correlated with the level of Unclassified_Ruminococcaceae

There was a negative correlation between *Unclassified_Ruminococcaceae* and C4 level but no significant correlation with ESR and C3 level. Other highly expressed differential floras had no relevance to ESR, C3 and C4 level (Table 5).

**Table 5:** The relevance of highly expressed differential floras and disease activity indexes in SLE group

	ESR		C3		C4	
	r	*P*	r	*P*	r	*P*
Unclassified_Lachnospiraceae	-0.227	0.350	-0.156	0.525	-0.175	0.473
Unclassified_Ruminococcaceae	-0.049	0.841	-0.407	0.084	**-0.494**	0.032^*^
Clostridium_XVIII	-0.239	0.325	-0.387	0.102	0.028	0.909
Streptococcus	0.019	0.937	-0.241	0.321	0.353	0.138
Bifidobacterium	0.130	0.595	-0.060	0.808	-0.181	0.457
Bacteroides	0.431	0.066	-0.027	0.912	0.056	0.819
Ruminococcus2	-0.248	0.306	-0.423	0.071	-0.245	0.311
Megamonas	-0.013	0.957	-0.331	0.166	-0.426	0.069

^*^P＜0.05

## Discussion

SLE, a common immune-mediated inflammatory syndrome, is characterized by over-activity of lymphocytes, production of autoantibodies and effects on multiple organs [10]. The abnormality of T cell subsets is the main pathogenesis of SLE [5]. Five major helper T cell subsets have been identified: Th1, Th2, Th17, Treg and Tfh cells, which coordinate the immune response via providing costimulatory signals and producing cytokines [11]. Th1 cells produce cytokines such as interferon (IFN)-γ, interleukin (IL)-2 and tumor necrosis factor (TNF) to prevent intracellular bacterial infection and delayed hypersensitivity. Th2 cells produce cytokines such as IL-4, IL-5, and IL-13 to enhance humoral immunity and eliminate worms and other extracellular pathogens, which are the drivers of pathological allergic reactions [12]. Th17 cells control the transcription of IL-17 to promote the recruitment and activation of neutrophils and eliminate extracellular bacteria [13]. Compared with healthy individuals, the levels of Th1, Th2 and Th17 cytokines in SLE patients are mostly increased and positively correlated with disease activity [12]. In contrast, Tregs produces cytokines such as transforming growth factor-β (TGF-β), IL-10 and IL-35 to inhibit antigen presentation and the activation and proliferation of T and B lymphocytes [14], and the number and functional defects of Tregs are closely related to SLE. Tfh cells are identified as a special subgroup of CD4^+^ T cells, which regulate the differentiation of B cells and the production of autoantibodies, and have been proved to be involved in the pathogenesis of SLE [15]. According to the expression of T cell receptor (TCR), T cells can be divided into two subsets (γδ T and αβ T cells). γδ T cells are a small number of T cells composed of γ and δ chains, which can recognize non-peptide antigens. It is widely recognized that γδ T cells may shift from typical Th1-like phenotype to Th2, Th17 and Tregs in a specific microenvironment [16]. In short, imbalance of T cell subsets leads to deregulated levels of cytokine and antibody, which may be one of the multiple factors in the pathogenesis and severity of SLE.

Numerous studies have emphasized the key role of gut microbiota in the pathogenesis of SLE, including the damage of intestinal mucosal barrier, molecular imitation of autoantigens and immune imbalance [17]. Gut pathogens with proinflammatory ability can reshape the immune environment by aberrant activation of innate immune and adaptive immune system [18]. Microbial antigens lead to the differentiation of inflammatory T cell subtypes such as Th17 cells, resulting in the upregulation of pro-inflammatory cytokines such as IL-17, IL-12 and IFN. Meanwhile, it inhibits Tregs differentiation and reduced transformation of anti-inflammatory cytokines such as TGF-β and IL-10. Recent findings have shown that the imbalance of Th17 cells and Tregs is a key feature of SLE disease activity [19, 20]. Gut pathogens can also trigger over-activation of B lymphocytes with the help of Tfh cells and produce pathogenic autoantibodies, which may affect the pathogenesis of SLE [21]. Interestingly, the imbalance of gut microbiota is not only the cause of autoimmunity, but also the result of autoimmunity [17]. Genetic mutations of TCR signal can initiate autoimmunity by promoting the positive selection of auto-reactive T cells and Tfh cells as well as the production of IgG. At the same time, these mutations reduce the positive selection of microbial reactive T cells and intestinal Tfh cells as well as intestinal IgA, leading to intestinal biological disorders, which further promotes the development of SLE. Therefore, gut microbiota imbalance, immune response, and inflammation are interrelated and jointly affect the occurrence and development of SLE.

According to recent research, it is increasingly clear that altered gut microbiome composition in SLE patients and murine models are closely related to disease activity [22, 23]. To better determine the role and mechanisms of gut microbiota in SLE, we compared 19 SLE patients and 16 HCs to characterize the gut microbiota pathophysiological changes in the progression of SLE. Compared with the HCs, the gut microbiome of SLE patients showed the following characteristics. (1) Microbial composition was changed. The distribution of OTU clusters exhibited that 342 OTUs were shared, 97 OTUs were specific to HCs and only 33 were specific to SLE group. (2) Immune disorders. The main upregulated T lymphocyte in SLE patients were Th17 cells and γδ T cells, and the main downregulated T lymphocyte were Tregs and Tfh cells. (3) The gut microbiome and lymphocyte were interrelated, and *Megamonas* may promote the progression of SLE, and *Bifidobacterium* and *Ruminococcus2* may improve the condition of SLE.

In this study, we observed that genus *Megamonas* was positively correlated with Th17 cells in SLE patients, and had no significant correlation with C3 and C4. Balmant et al. explored the difference of gut microbiome between inactive SLE women using hydroxychloroquine (HCQ) and healthy women. *Megamonas* is abundant in SLE females and negatively regulates C3 complement levels [24]. Additionally, a study had evaluated the association between bladder microbiome and urine metabolism, cytokines as well as disease phenotype in active patients SLE, and reported a negative correlation between the abundance of *Megamonas* and C3 complement levels [25]. At the same time, the abundance of *Megamonas* in primary Sjogren's syndrome patients was significantly more than HCs, and positively correlated with clinical disease indexes [26]. In addition, in patients with non-rheumatic diseases, *Megamonas* is associated with pro-inflammatory cytokines [27]. These findings suggest that *Megamonas* plays an important role in the disease activity of SLE.

Our results showed that *Ruminococcus2* was positively correlated with the absolute number of Tregs, while *Bifidobacterium* was negatively correlated with Th1 cells. Some studies have also demonstrated that *Ruminococcus2* was deficient in autoimmune diseases [28]. The insufficiency of *Ruminococcus2* may impair immune tolerance of Tregs, which indicated *Ruminococcus2* may be one of the microbes that can elicit a protective response to SLE. It is reported that *Bifidobacterium* decreased in the feces of SLE, which was negatively correlated with disease activity. Supplementation of *Bifidobacterium* can prevent over-activation of CD4 + lymphocytes and reconstruct the Treg/Th17/Th1 imbalance associated with gut microbiome [29]. Coincidentally, a study has demonstrated that some anti-inflammatory bacteria such as *Bifidobacterium* reduced in SLE patients, resulting in the release of inflammatory factors and increased level of systemic inflammation [30]. Not only that, short-term intervention of gut microbiota before onset can affect the treatment of prednisone in SLE mice, and the decrease of *Bifidobacterium* abundance may reduce therapeutic efficiency of prednisone [31]. Therefore, the regulation of gut microbiota may affect the severity, progress, and treatment of SLE, which can be used as important reference for the choice of clinical treatment of SLE.

Compared with the analysis of single microbiome dataset, our study provides additional insights by immune cell subpopulation analysis, which demonstrated that the gut microbiome and cell subsets were changed and interconnected in SLE patients compared with HCs. These associated gut microbiota of SLE are worthy to further research and development, which may provide a new idea for SLE treatment.

### Supplementary Information

Supplementary Material 1.

## Data Availability

Our SRA records will be accessible with the following link: https://www.ncbi.nlm.nih.gov/sra/PRJNA1085098 Or PRJNA1085098.

## Abbreviations

SLE: Systemic Lupus Erythematosus
SFB: Segmented Filamentous Bacteria
Th17: Type 17 T helper
Tregs: T regulatory cells
ACR: American college of Rheumatology
EULAR: European League Against Rheumatism
HCs: Healthy Controls
ESR: Erythrocyte Sedimentation Rate
C3: Complement 3
C4: Complement 4
IFN-γ: Interferon-γ
IL-2: Interleukin-2
TNF: Tumor Necrosis Factor
TGF-β: Transforming Growth Factor-β
TCR: T Cell Receptor
HCQ: Hydroxychloroquine
