# Supplementary material for: Gut microbiota regulation of T lymphocyte subsets during systemic lupus erythematosus
Source: BMC Immunol. 2024 Jul 8;25:41. doi: 10.1186/s12865-024-00632-0 (PMC11229189; doi:10.1186/s12865-024-00632-0)
Supplement: Supplementary file 1 — Supplementary Material 1. [file 12865_2024_632_MOESM1_ESM.pdf]

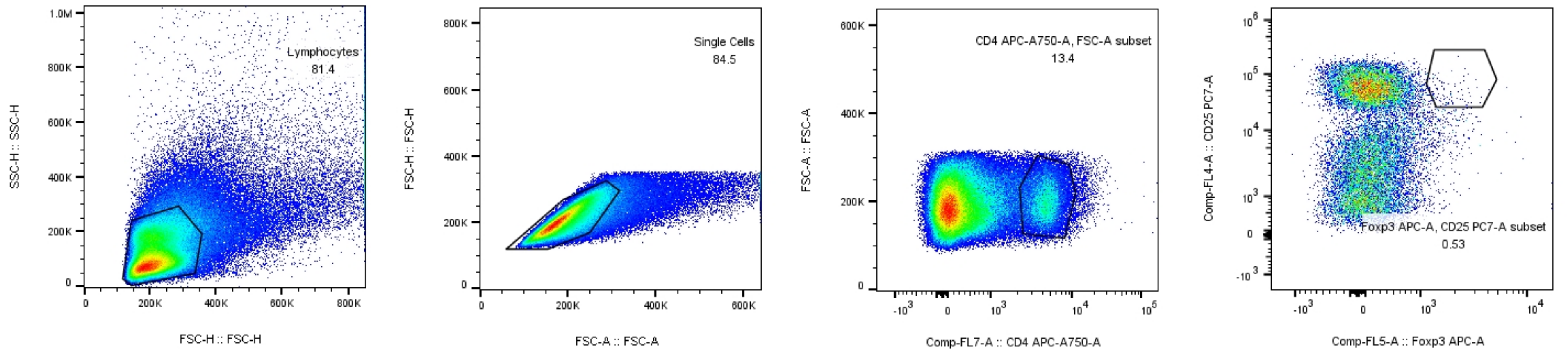

Figure S1: Flow graph of Tregs in SLE patients

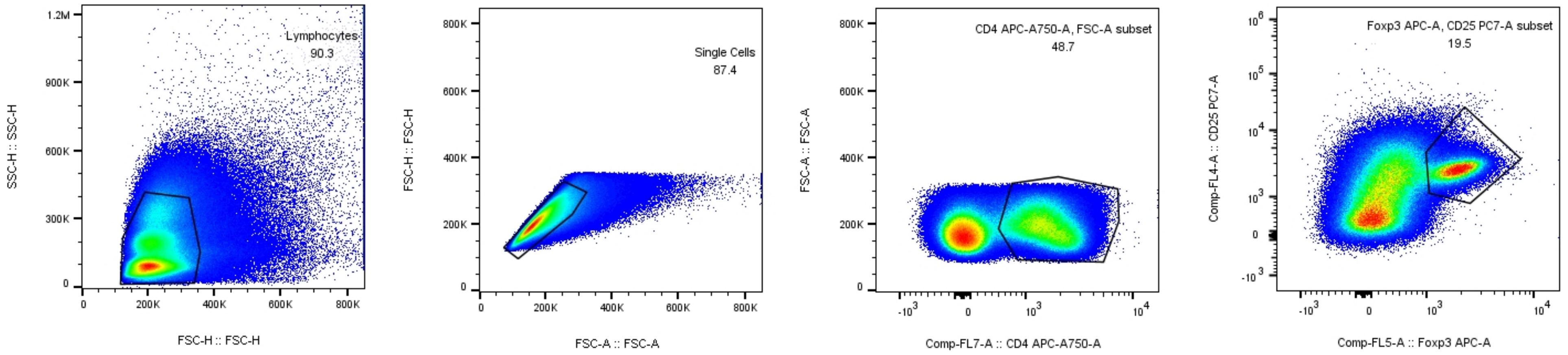

Figure S2: Flow graph of Tregs in healthy controls

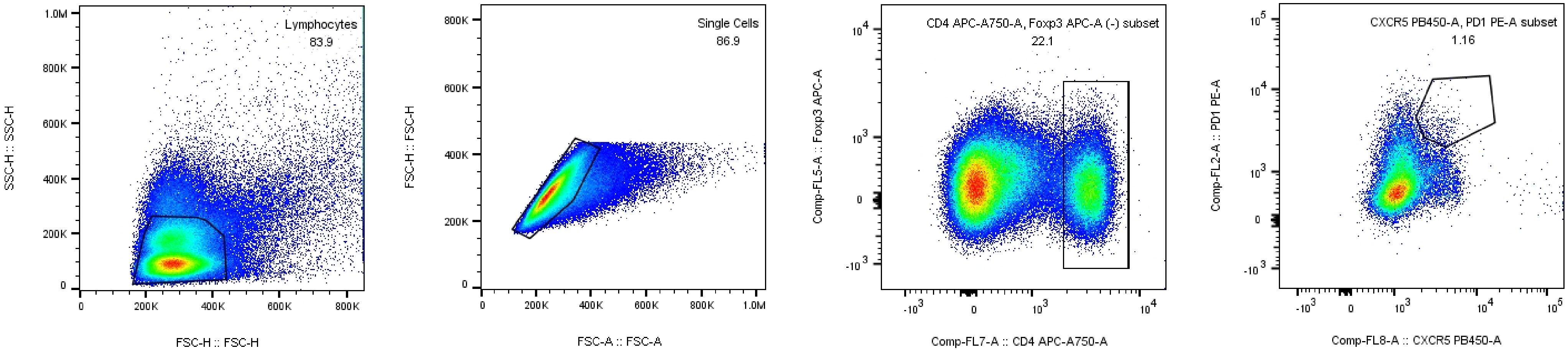

Figure S3: Flow graph of Tfh cells in SLE patients

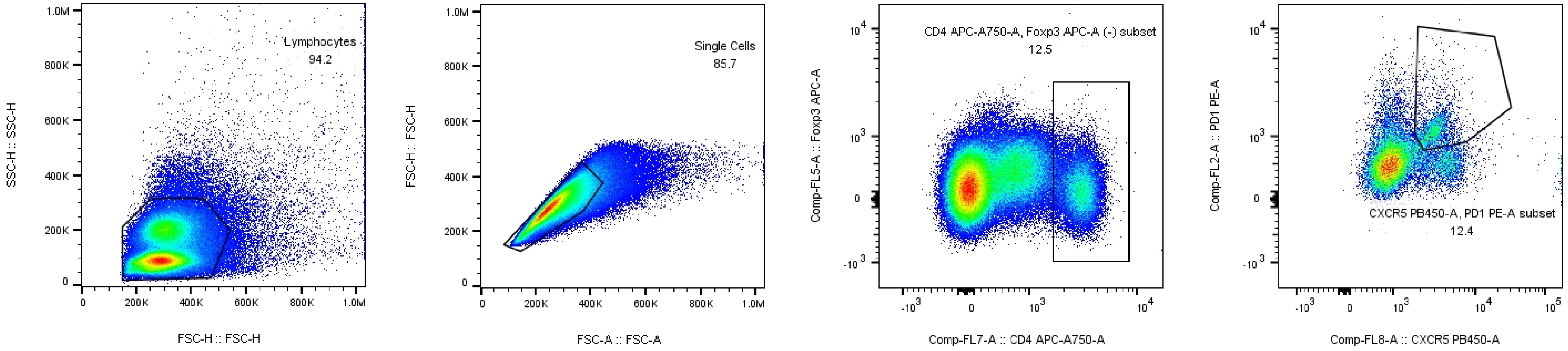

Figure S4: Flow graph of Tfh cells in healthy controls

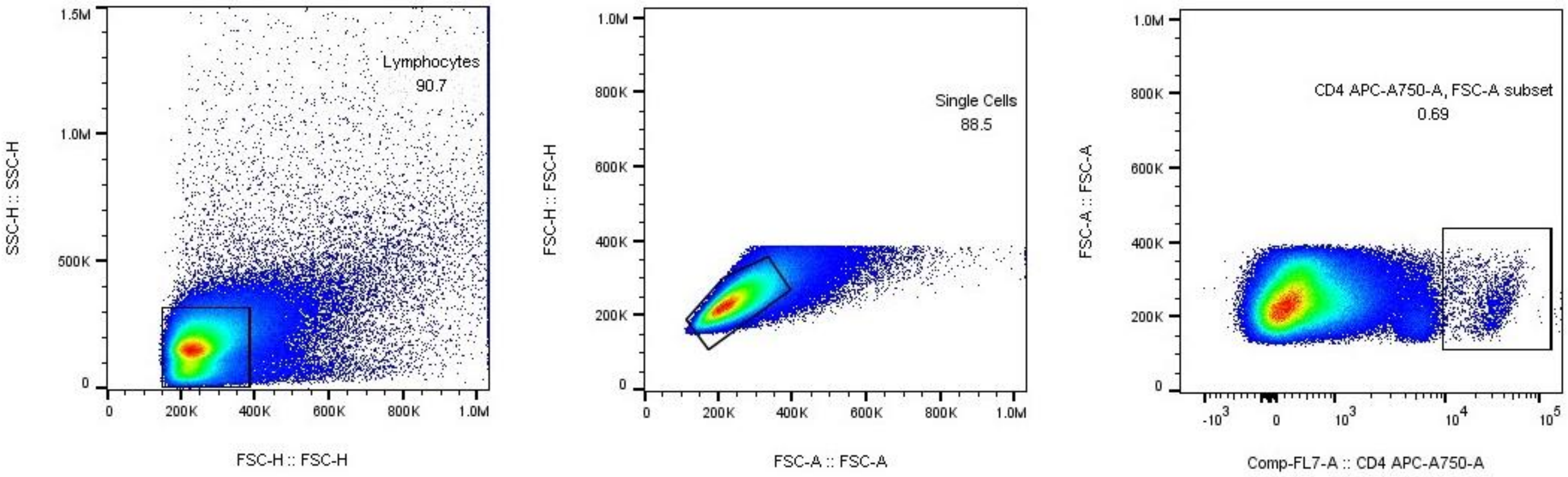

Figure S5: Flow graph of Naïve CD4+T cells in SLE patients

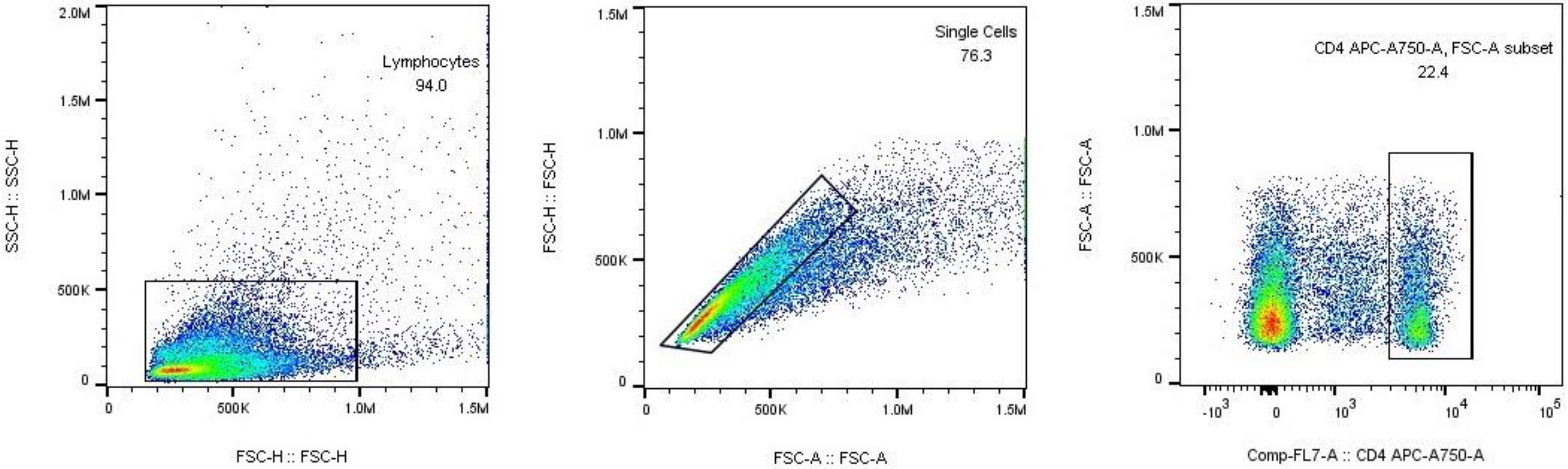

Figure S6: Flow graph of Naïve CD4+T cells in healthy controls

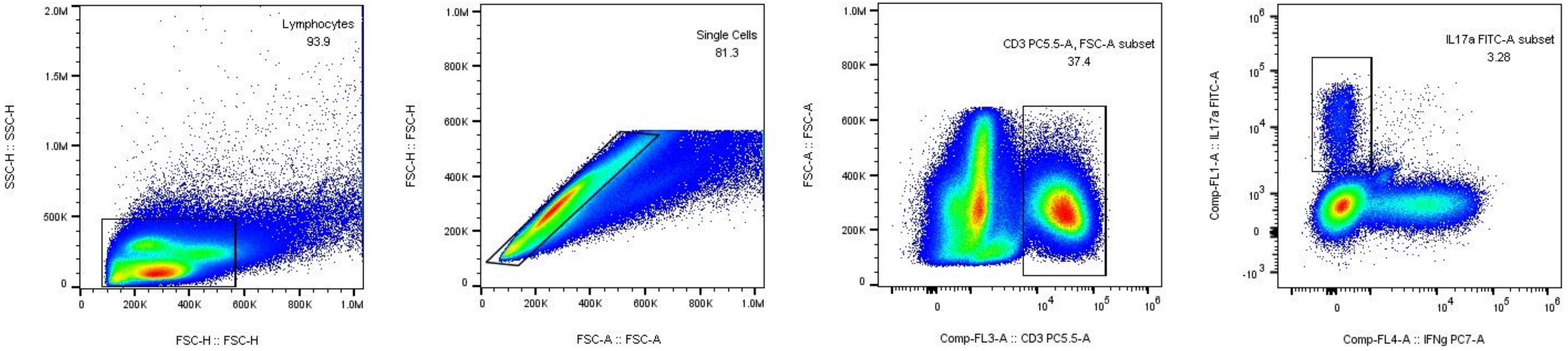

Figure S7: Flow graph of Th17 cells in SLE patients

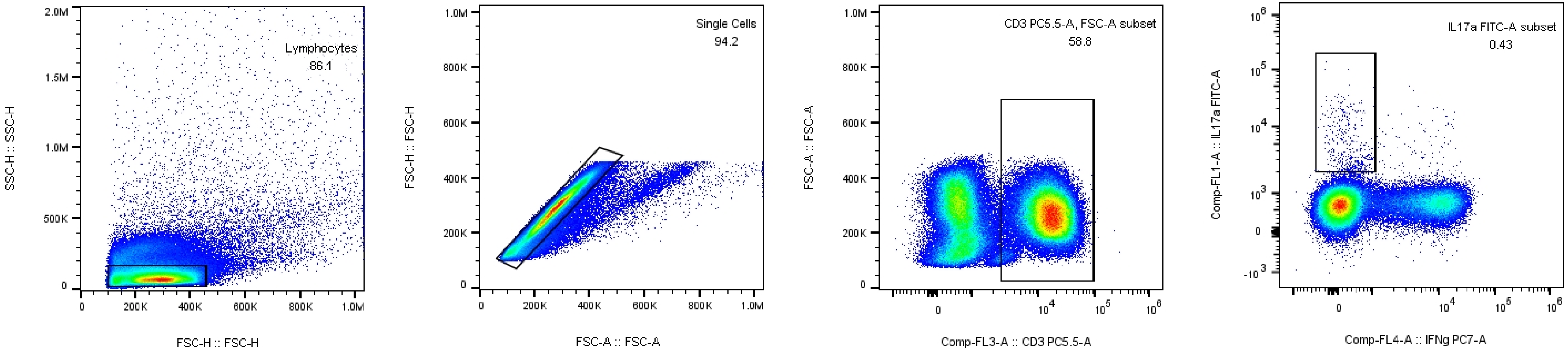

Figure S8: Flow graph of Th17 cells in healthy controls

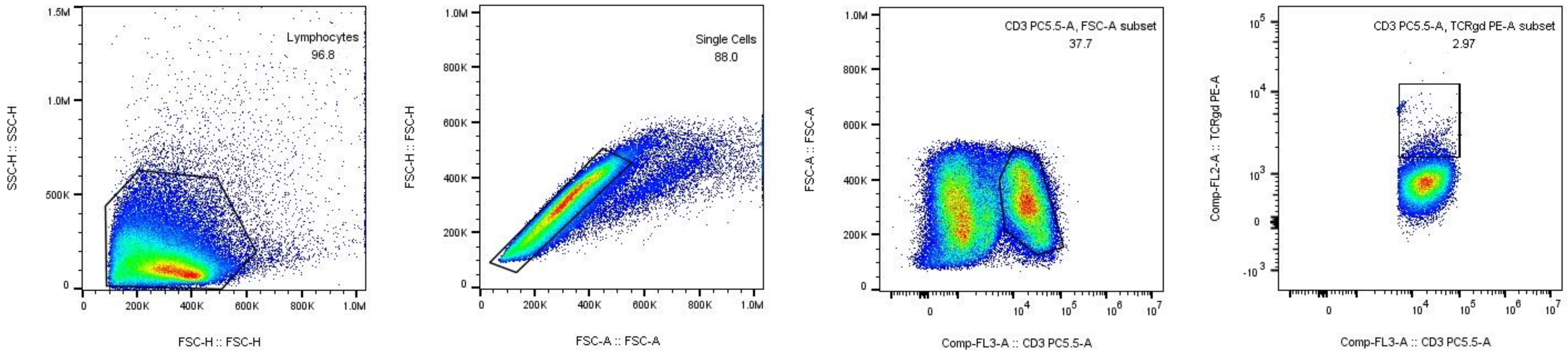

Figure S9: Flow graph of γδ T cells in SLE patients

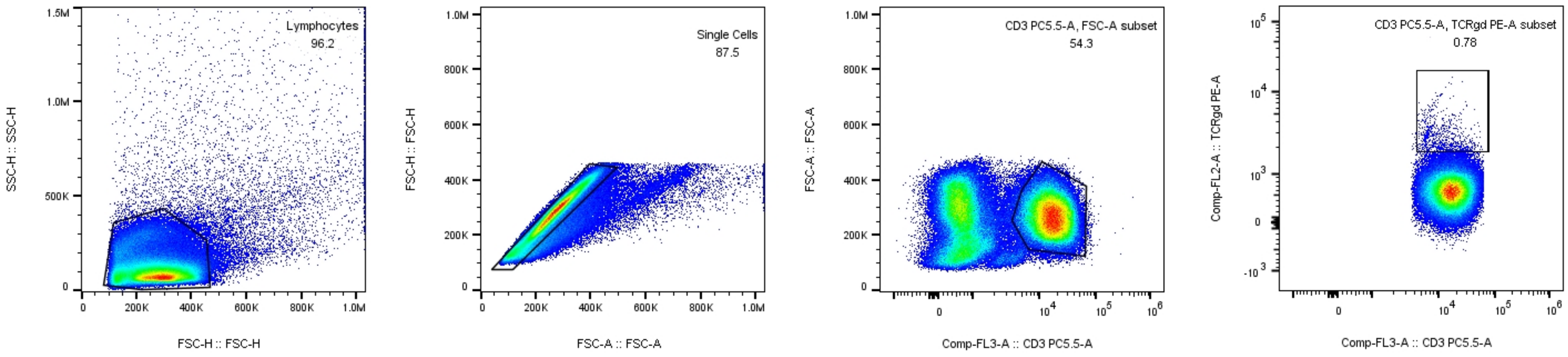

Figure S10: Flow graph of γδ T cells in healthy controls
